# Supplementary material for: Dendritic integration in olfactory bulb granule cells upon simultaneous multispine activation: Low thresholds for nonlocal spiking activity
Source: PLoS Biol. 2020 Sep 23;18(9):e3000873. doi: 10.1371/journal.pbio.3000873 (PMC7535128; doi:10.1371/journal.pbio.3000873)
Supplement: S1 Fig — a: Blue histogram: amplitude distribution of single-spine uEPSPs in this study (n = 272 spines). Mean uEPSP: 1.4 ± 1.4 mV. Gray histogram: distribution of uEPSPs from previous study (Bywalez and colleagues 2015; right axis, n = 47 spines). b: Recordings of multiple uEPSP responses from the same spine (n = 9 ± 3 responses on average, n = 18 spines, mean uEPSP 1.6 ± 1.0 mV) from Bywalez and colleagues 2015, analyzed for their SD. Highly significant correlation (p < 0.001), linear fit shown. For the mean value of uEPSPs in the current study of 1.4 mV, SDsingle-spine is thus on the order of 0.4 mV (blue arrows). c: Extrapolation of multispine EPSP amplitudes versus the arithmetic single-spine EPSP sum for linear summation from b and the same mean single uEPSP and SDsingle-spine response for all spines (as extrapolated from b). Variations of mean EPSP size across spines were not taken into account because these should not influence the linearity of summation. White numerals: respective spine number. Error bars in the x-dimension (arithmetic sum): Black: Expected standard deviation SDsum for ideal recording conditions (at least 6 stimulations per spine, SDsum = (√Nspines)*SDsingle-spine, see Methods). Blue: standard deviation of EPSP amplitudes in our data set extrapolated from the ideal SD. Because there are only 2 stimulations per spine instead of the 6 stimulations required to properly measure SDsingle-spine, the actual SDsingle-spine is increased by a factor of √3 compared with the ideal SDsingle-spine and thus the SDsum is also increased by a factor of √3 (see Methods). Error bars in the y-dimension (compound EPSP): Black: Expected standard deviation SDmulti-spine for ideal recording conditions and linear summation of similar uEPSPs (at least 6 stimulations per spine set, SDmulti-spine = (√Nspines)*SDsingle-spine, see Methods). Blue: SD of EPSP amplitudes in our data set extrapolated from the ideal SD, similar to the x-dimension: since there are only 2 stimulations p [file pbio.3000873.s001.docx]

**
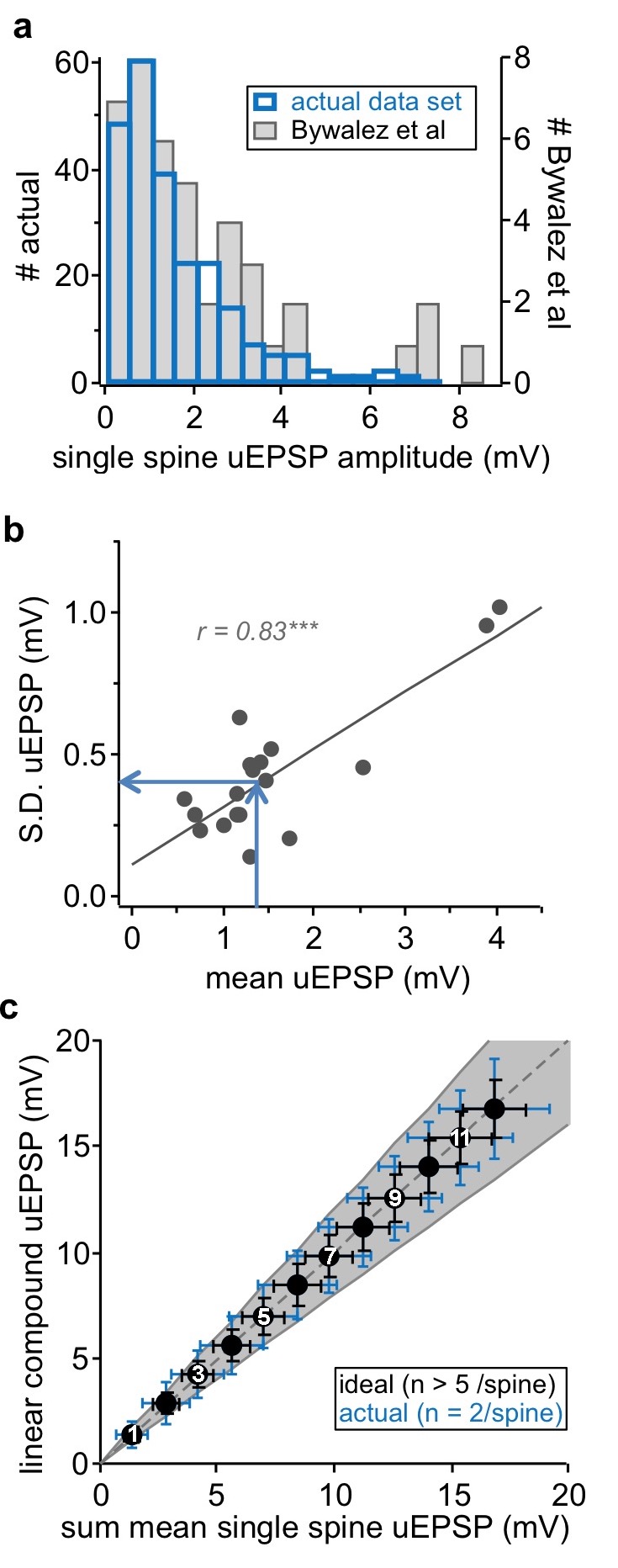
**

**Fig S1. Distribution of single uEPSP amplitudes, variance of single uEPSPs and estimate of variance for compound and summated uEPSPs**

**a:** Blue histogram: amplitude distribution of single spine uEPSPs in this study (n = 272 spines). Mean uEPSP: 1.4 ± 1.4 mV. Grey histogram: distribution of uEPSPs from previous study (Bywalez et al. 2015; right axis, n = 47 spines).

**b**: Recordings of multiple uEPSP responses from the same spine (n = 9 ± 3 responses on average, n = 18 spines, mean uEPSP 1.6 ± 1.0 mV) from Bywalez et al. 2015, analysed for their standard deviation (SD). Highly significant correlation (p < 0.001), linear fit shown. For the mean value of uEPSPs in the current study of 1.4 mV, SD_single-spine_ is thus on the order of 0.4 mV (blue arrows).

**c:** Extrapolation of multi-spine EPSP amplitudes versus the arithmetic single-spine EPSP sum for linear summation from b and the same mean single uEPSP and SD_single-spine_ response for all spines (as extrapolated from b). Variations of mean EPSP size across spines were not taken into account since these should not influence the linearity of summation. White numerals: respective spine number.

Error bars in the x-dimension (arithmetic sum): Black: Expected standard deviation SD_sum_ for ideal recording conditions (at least 6 stimulations per spine, SD_sum_ = (√N_spines_)*SD_single spine_, see Methods). Blue: standard deviation of EPSP amplitudes in our data set extrapolated from the ideal SD: since there are only two stimulations per spine instead of the six stimulations required to properly measure SD_single-spine_, the actual SD_single-spine_ is increased by a factor of √3 compared to the ideal SD_single-spine_ and thus the SD_sum_ is also increased by a factor of √3 (see Methods).

Error bars in the y-dimension (compound EPSP): Black: Expected standard deviation SD_multi-spine_ for ideal recording conditions and linear summation of similar uEPSPs (at least 6 stimulations per spine set, SD_multi-spine_ = (√N_spines_)*SD_single spine_, see Methods). Blue: Standard deviation of EPSP amplitudes in our data set extrapolated from the ideal SD, similar to the x-dimension: since there are only 2 stimulations per spine set, the actual SD_multi-spine_ is increased by a factor of √3 compared to the ideal SD_multi-spine_.
