# Supplementary material for: Dendritic integration in olfactory bulb granule cells upon simultaneous multispine activation: Low thresholds for nonlocal spiking activity
Source: PLoS Biol. 2020 Sep 23;18(9):e3000873. doi: 10.1371/journal.pbio.3000873 (PMC7535128; doi:10.1371/journal.pbio.3000873)
Supplement: S2 Fig — Individual data points from paired data comparisons across threshold for Ca2+-spikes (a), D-spikes (b) and effect of TTX on D-spike transitions (c). These data were not plotted in the main figures for sake of clarity. In a, b data are shown normalized to the average value below threshold (except for ΔF/F dendrite because of several points with value zero) and corrected for linear trend in subthreshold data (see Methods). In c, changes Δ in parameter values across threshold in TTX are shown normalized to their increase Δ in control, thus no correction for linear trends is required. Analysis of half duration is missing because there were not enough data points for statistical analysis. *p < 0.05, **p < 0.01, ***p < 0.001. D-spike, dendritic Na+-spikes; TTX, tetrodotoxin. (DOCX) [file pbio.3000873.s002.docx]

**Supporting Information Table S1**

**Table S1. Robustness of supralinearity criterion O/I ratio ≥ 1.2**

| **Parameter** | **Value O/I ratio** | | | **Normalized to 1.2** | |
| --- | --- | --- | --- | --- | --- |
|  | **1.1** | **1.2** | **1.3** | **1.1** | **1.3** |
| **Number of supralinear cells** | 18 | 18 | 17 | 1 | 0.94 |
| **D-spike threshold spine number** | 5.7 | 6.7 | 6.7 | 0.85 | 0.99 |
| **∆ EPSP amp. at threshold (mV)** | 2.8 | 3.9 | 4.4 | 0.72 | 1.13 |
| **∆ O/I ratio at threshold** | 0.69 | 0.74 | 0.80 | 0.93 | 1.08 |
| **∆ rate of rise at threshold (V/s)** | 1.1 | 1.1 | 1.3 | 0.99 | 1.19 |
| **∆ rise time at threshold (ms)** | 6.5 | 8.8 | 8.2 | 0.74 | 0.93 |
| **∆ ∆F/F spine 1 at threshold (%)** | 62 | 71 | 64 | 0.87 | 0.09 |

The criterion was varied by ± 0.1 and the respective data of the individual cells were rearranged accordingly before averaging.
