# Supplementary material for: Dendritic integration in olfactory bulb granule cells upon simultaneous multispine activation: Low thresholds for nonlocal spiking activity
Source: PLoS Biol. 2020 Sep 23;18(9):e3000873. doi: 10.1371/journal.pbio.3000873 (PMC7535128; doi:10.1371/journal.pbio.3000873)
Supplement: S1 Table — The criterion was varied by ± 0.1 and the respective data of the individual cells were rearranged accordingly before averaging. O/I, output/input. (DOCX) [file pbio.3000873.s003.docx]

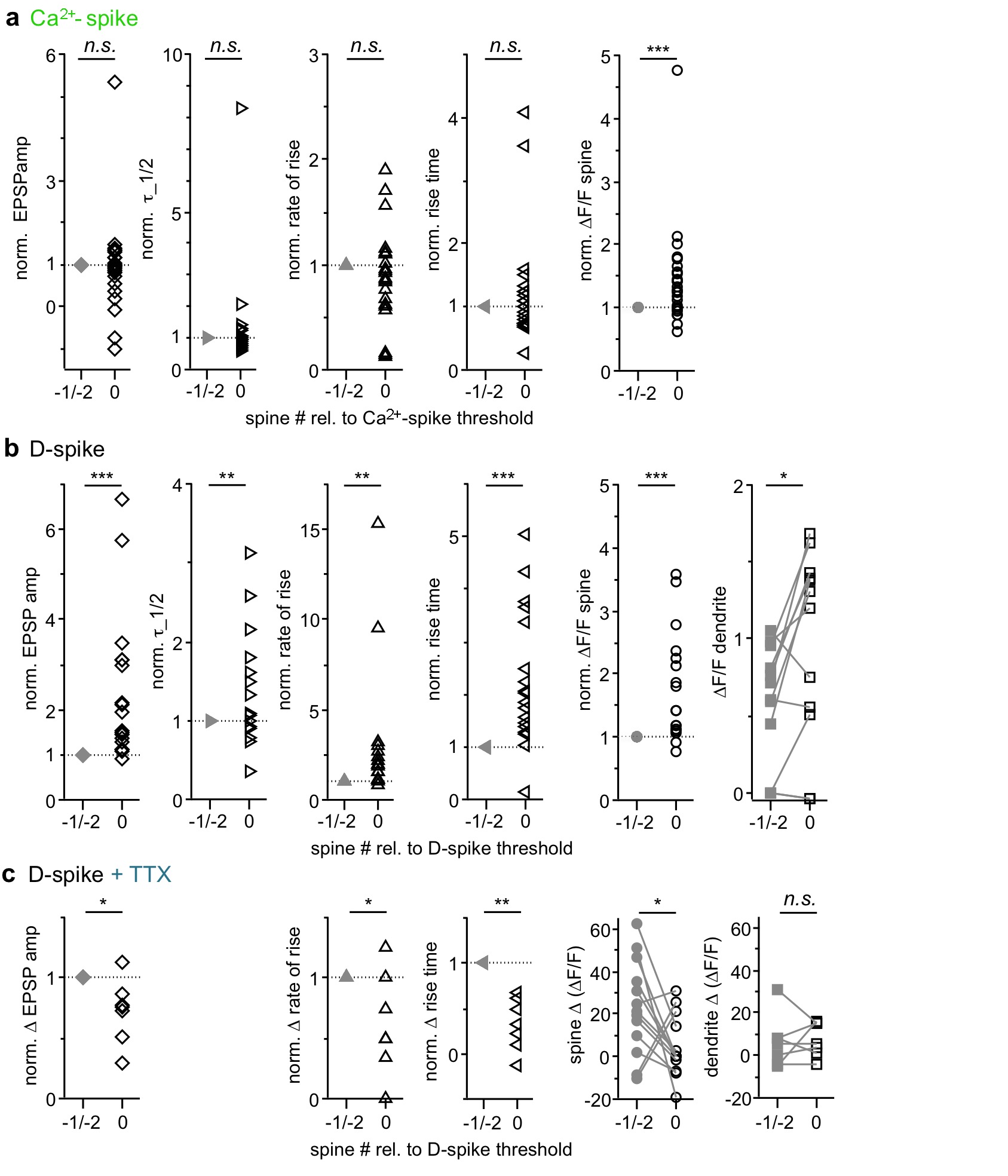


**Fig S2.** **Individual data sets at threshold for Ca^2+^-spike and D-spike**

Individual data points from paired data comparisons across threshold for Ca^2+^-spikes (**a**), D-spikes (**b**) and effect of TTX on D-spike transitions (**c**). These data were not plotted in the main figures for sake of clarity. In **a**, **b** data are shown normalized to the average value below threshold (except for ∆F/F dendrite because of several points with value zero) and corrected for linear trend in subthreshold data (see Methods). In **c**, changes ∆ in parameter values across threshold in TTX are shown normalized to their increase ∆ in control, thus no correction for linear trends is required. Analysis of half duration is missing because there were not enough data points for statistical analysis.

* : p < 0.05, ** : p < 0.01, *** : p < 0.001
