# Supplementary material for: Dendritic integration in olfactory bulb granule cells upon simultaneous multispine activation: Low thresholds for nonlocal spiking activity
Source: PLoS Biol. 2020 Sep 23;18(9):e3000873. doi: 10.1371/journal.pbio.3000873 (PMC7535128; doi:10.1371/journal.pbio.3000873)
Supplement: Attachment — Submitted filename: ResponseToReviewers.docx [file pbio.3000873.s004.docx]

*Before answering to the comments point by point, we wish to thank the reviewers for their detailed suggestions and useful input. We also wish to apologize for the overly abridged presentation of the complex material in the original version of this manuscript, which was in part due to our efforts to adhere to article formats and space limitations imposed by other journals. To improve accessibility, we have extended the manuscript in various ways as evident from the track-changes version, most importantly by adding a part on dendritic integration in general to the introduction and a graphical summary of our main findings (new Fig. 9) and by ensuring a better coherence between text flow and figure succession. Additional citations are listed at the end of this response.*

REVIEWS:

**Reviewer #1, Diego Restrepo:** The manuscript by Muller and Egger presents a study of the non-linear integration of multi spine activation in granule cells of the olfactory bulb. The authors use a novel holographic stimulation method to compare activation at will of subsets of spines separately or simultaneously. This is a novel approach that yields interesting data that is potentially of interest, not only to those understanding olfactory function, but to the broader readership interested in mechanisms of non-linear integration in dendrites relevant to neural function processing in areas such as cortex, hippocampus and cerebellum. My enthusiasm is diminished significantly by the deficient presentation of the data and the lack of validation of the parameters used to classify supralinear changes in the single spine EPSP sum (suEPSP) vs. the compound multiple spine EPSP (cuEPSP).

Major criticisms

1. The presentation of the data is not clear and is currently not suitable for the broad readership of PLoS Biology. In particular, the data are presented in a manner that is difficult to follow. For example, presentation of the section on transition to supralinear behavior with D-spikes (starting in line 212) is confusing. First, the authors start by discussing Fig. 3c, without an introduction to the D-spine (Figs. 3a and b are not mentioned in the text). Second, the wording is confusing (e.g. please re-word the sentence starting with "Since Ca2+-spike…" in line 213). Third, it does not help that in most panels there is not a label of what the different colors/symbols mean (e.g. what is green vs black in Fig. 3b?). I find these problems throughout the results. Please revise the flow of the text in the results and presentation of all the figures. Please refer to all panels in the text and cite the panels in alphabetical order (if possible).

*To improve access for a broader readership, we have now added a general introduction (l.38-57; also requested by reviewer 3) in which the different spike types are introduced. We have aligned the order of figure panels and the Results text and made sure that every panel is mentioned in the text. The only exception from the alignment is the part on D-spike pharmacology (Na_v_/TTX) that we briefly mention in advance because of its importance for proving the identity of the D-spike.*

*We have rephrased and clarified the introduction to the section on D-spikes (l. 268- 274).*

*We also have streamlined the use of color labels for spike types and added legends within figures throughout.*

2. The criterion for classification of supralinearity at 0.2 is arbitrary. The authors provide a justification in the methods, but I am not convinced this is a well-justified choice. Please present data indicating why this criterion was chosen. Is there a statistical approach that could justify the choice (e.g. bootstrap prediction of the cuEPSP at the higher number of spines using the results for the first few spines?). Or, what happens when the arbitrary criterion is varied? Incidentally, please provide a more comprehensive introduction of quantification of integration and the supralinear criterion in the results.

*With regard to the choice of the supralinearity criterion, we were aware of this issue prior to the first submissio. We had performed an intense search in the existing literature and discussed the choice with an expert in the field who also did not have a ready solution.*

*We agree however that the explanation of the criterion is unsatisfactory and have now attempted to further substantiate the criterion along the lines indicated by the reviewer. To this end, we have added a new supporting figure (now S1), that explains the estimate, and a supporting table 1 that provides the requested results of variation of the criterion. In the figure, we first show the distributions of single uEPSP amplitudes of our current and our previous study (Fig. S1a, Bywalez et al. 2015) where single spines were stimulated more often with uncaging than in the present study (the single uEPSP amplitude data were also asked for in Minor point 3 by the same reviewer). Then we show data from the previous study that indicate the amount of variance inherent to our stimulation technique (unpublished so far, Fig. S1b; data are now provided at the respository) and use this information to predict the variances of the compound and summated uEPSPs in the case of linear summation for the actual situation in our experimental set where the number of measurements per spine is actually just n = 2 due to technical limitations (Fig. S1c). This prediction is now explained in more detail in the Methods section (l. 882 ff) and also in the legend of Fig S1c. According to this coarse estimate, our criterion should be sufficiently strong to correctly classify our data for spine numbers from 6 onwards, which roughly matches the onset of supralinear summation in our data set (6.5 ± 2.7 spines).*

*The requested results of the variation of the criterion (from output/input O/I ratio 1.2 to 1.1 and 1.3, respectively) are listed in the new Supporting Table 1, showing most importantly that the total number (and identity) of cells classified as supralinear will not change if the criterion is lowered to 1.1, as well as the change in the rate of rise at threshold, which is indicative of D-spikes. Thus the criterion is robust with regard to the differentiation between supralinear and linear cells and the detection of D-spikes. This finding is now also included in the Results section, along with a reference to the supporting material (l. 167 -171.).*

*It should be noted however that at this point we cannot discriminate the biological variance between single EPSPs from the variance introduced by the uncaging technique. Thus the criterion is specific to both our experimental technique and amount of sampling and thus would need to be rederived for other situations. Also, the above analysis is just a rather coarse estimate, but, as proposed by the reviewer, the variation of the criterion allows to test its robustness.*

*We also tested whether for the cells that did not classify as supralinear according to the criterion there was any missed correlation, by aligning their data to their largest increase in O/I ratio and checking for a concomitant increase in rate of rise, ∆F/F, or in rise time. No such effects were observed.*

3. The authors are well-versed in dendritic processing, and I enjoyed reading the discussion. However, the section on functional implications needs reworking. First, the text indicates that in vivo GCs firing is sparse (line 639). This is correct in anesthetized animals, but it is very different in the awake animal (Cazakoff et al. 2014). Second, there needs to be a better discussion of what this would mean in terms of phase amplitude coupling in awake animals (see Youngstorm and Strowbridge 2015, Zhouang et al 2019) that has been recently implicated in conveying contextual identity (Losacco et. al. 2020).

*Thanks for adding these aspects! We have extended the respective paragraph (l. 747-758), with the only exception that we did not wish to cite the Zhouang et al paper, since its findings seem in part to be at variance with our own observations in the semi-intact nose brain preparation where there is no respiration (Perez et al. 2015, unpublished observations).*

Minor issues

1. For a reader unfamiliar with the different modes of excitability it is difficult to follow the denomination of the spikes. Please revise the definition of the different modes (e.g. D-spikes) in the introduction (line 41) to make it absolutely clear what these are, and use the same nomenclature throughout. Maybe an introductory figure would help?

*The introduction has been revised and extended (l.38-57), also in response to point 2 by reviewer 3. The denomination of the spikes has been streamlined throughout the manuscript and is now also further illustrated in the new graphical summary (Fig. 9).*

2. Lines 33-36. Please provide a citation for the sentence claiming within glomerular column communication of M/Ts (do the authors mean gap junctions and/or juxtaglomerular inhibitory interneuron connections?).

*We were referring to direct intraglomerular interactions between mitral/tufted cell apical dendritic tufts; this has been clarified and references have been included in l. 65-66.*

3. Lines 87-89. Please show histograms for the number of APs with maximal stimulation and for the average suEPSP.

*The histogram for single uEPSPs is now shown in Supporting Fig S1a; since the number of APs was not broadly distributed and also often varied across stimulations of the same spine set, we did not add a histogram for these but extended the respective text in the Results, as well as the information on spike latency and single uEPSP amplitude distribution in l. 123-130.*

4. The authors should always provide the number of observations when they present a statistical estimate, or test. Please revise throughout (e.g. in line 123 the average, and SD? Is presented for the number of spines for supralinearity without stating the n). Please give the n whenever a statistical test/value is presented.

*We have fixed these omissions.*

5. Please give the statistics for the statement in lines 268-270.

*These statistics are given in the respective figure panel caption (now Fig. 5). To improve the readability of the Results section, we have given the details for statistics in the figure captions whenever possible.*

6. Line 441. Please state the specificity of mibefradil (L vs T type) at the concentration used.

*This information has been inserted at l. 510: T-type Ca_v_s IC_50_ 2.7 µM versus 18.6 µM for L-type Ca_v_s, Mehrke et al. 1994*

7. Figs. 2d,e. The horizontal bars denoting statistical significance are misplaced? Also, was there a correction for multiple comparisons?

*As explained in the Methods section (part on Statistics, l. 951-967), we had to devise a method to compare data across thresholds because of differing numbers of data points which involves including data from both -1 and -2 spines below threshold (therefore the left straight bar). This method is referred to at both the first occurrence of such threshold data in the manuscript (l. 260 f.) and in the legend of Fig. 2 where it was applied for the first time (l. 236). The individual data points for all these comparisons across thresholds are shown in Supporting Fig. S2.*

*For this analysis, there was no correction for multiple comparisons, since these statistics concerned only the behavior at threshold. However, we corrected for the basic linear trend in the data (see Methods l. 963 f.). The individual data points were omitted for sake of clarity from the main figure panels but are shown (with linear correction) in Fig. S2. Corrections for multiple comparisons were used throughout for the pharmacology data (Fig. 5-7).*

8. For Fig. 3d, right panel there are no asterisks. Are the differences not significant? Also see Fig. 4e.

*Since an increase of the O/I ratio beyond 1.2 was used as criterion for the onset of the D-spike and thus for aligning the data, the difference was not tested for significance in the original manuscript. The panel has now its own label 3e. Similarly in Fig 4e: the increase in dendritic ∆F/F above noise level was used as a criterion for the onset of the Ca^2+^ spike and thus its significance was not tested for. The reason for the missing significance ratings is now mentioned explicitly in the figure legends of both Fig. 3e (l.301 f) and 4e (l. 346 f.).*

9. Please provide error bars for Fig. 4b.

*In Fig 4b and c, error bars (SD) have been included and the linear fit was removed, in line with the second comment by reviewer 2.*

Reviewer #2: In this manuscript, Mueller and Egger describe the physiological signatures of three types of nonlinear events observed in granule cells of the olfactory bulb. Using simultaneous uncaging afforded by holographic light presentations, they compare how compound stimulations differs from individual stimulations, and thus characterise and quantify the phenomena they observe. The study reveals incremental but step-like activations of low threshold Ca2+ spikes, dendritic spikes, and ultimately a more global Na+ spike, as well as identifying which channels are involved by additional pharmacology. Their results also suggest that, unlike larger neurons found elsewhere in the brain, for these neurons, the input locations or the morphological constraints correlate less with the likelihood of generating Na+ and Ca+ spikes. The strength of this study also comes from simultaneous observations of somatic electrophysiological signals and the dendritic Ca2+ signals, as the two types of information can reinforce our understanding of the events that are described. Overall, the study is important, rigorous, and the data are of high quality.

The manuscript, however, should be written more accessibly. One reason why this manuscript was extremely difficult to read is the use of many abbreviations. Some abbreviations are common and thus easily understood (names of channels, commonly accepted physiological abbreviations, such as EPSP, Na+ spikes etc). However, others (e.g., sO/I, S1, TPU, SuEPSP/CuEPSP, D-spike, LTS etc etc) are less common. As some of these do not appear frequently in the manuscript, please consider spelling out, and keep abbreviations only to those that are essential.

*We have eliminated all the abbreviations that were used less frequently (S1, TPU, suEPSP/cuEPSP, LTS) or that hamper the flow of the text such as GC. We have kept D-spike and sO/I because ‚subthreshold output/input relationship’ is rather unwieldy and these abbreviations are also needed to keep axis captions in the figure panels short.*

Another stylistic aspect that made reading of this manuscript difficult is a slightly convoluted narrative in a few places. For example, when describing the results about response amplitudes that precede a full-blown Na^+^-spike (page 17), in paragraph 1, the authors describe what "the dendrites know about", only to negate what they write in the last paragraph of the same page. While possibly this could have been the history behind the ultimate interpretations, it is better to state just the latter so as not to confuse readers (unless it is in reference to an earlier study).

*This is an excellent point and the manuscript has been reworked accordingly, including the removal of the linear fits from Fig. 2b. To further improve the flow of the text, we have also aligned the figure panels and the Results (see also response to Reviewer 1 and response to the next point).*

Related to the above points, figures 2-4 mainly describe inputs required for the recruitment of each type of nonlinear events. However, each figure also has a small section on all three types of events. This back-and-forth makes it a bit confusing for readers. In the grand scheme of things (compared to the main points presented in this manuscript), there seems to be too much detail on the observation that, for each type of active events, there is a sudden increase in the Ca^2+^ signal.

*As stated above, we have now attempted to remove such back-and-forth as far as possible (see also response to reviewer 1). We hope to have amended at least part of this problem by reassembling all figure panels with TTX pharmacology (in former Fig 3,4,5) into one Figure (Fig. 5) and rewriting the text along this new order.*

*As to the step-like increases in Ca^2+^ signals, we think that the impression of an over-emphasis of this observation might have arisen because we included the statistics for these observations in the text whereas statistics for other findings are often described in the figure legends. Still, the respective Results section (l. 320 ff) is now more concise than in the original version because of the more stringent rewriting along the line proposed above by the same reviewer.*

In the data presented in Fig. 6, observations from the control vs. mibefradil conditions differ between panels d vs. g. What is the difference?

*The difference is that the lower panels contain a smaller set of experiments (where Cadmium was applied after mibefradil to check for HVA Cavs). The respective n numbers of experiments are given in the legend. We now explicitly mention in the Results that these experiments were done in a subset of cells and introduced an extra paragraph for them (l. 556-560).*

Minor comments -

Fig. 3b: Please provide a brief explanation for the scale bar -

*The confusing shared scale bars between panel 3b and 3f no longer exist, since 3f has been moved to Fig 5.*

Fig. 4g (left panel): The lines used for the TTX data are hard to see. Please increase the contrast.

*In the revised figures, all recordings in the presence of TTX are color coded (new Fig. 5).*

Reviewer #3: In this manuscript, Egger and colleagues use focal, patterned photorelease of glutamate (to activate multiple single spines) of olfactory bulb granule cells to investigate how these cells integrate synaptic information across their dendritic tree. Dendritic integration is a fundamental problem/phenomenon in neuroscience. Olfactory bulb granule cells are particularly intriguing for many reasons - they are inhibitory neurons with dendritic spines, they lack axons, they release transmitter from dendrites (often from reciprocal synapses) and they have local and global processing modes that include nonlinear events mediated by Ca and/or Na. This study covers all these points! The authors demonstrate that activating a small number of spines (synapses) suffices to generate nonlinear events with interesting features that can be tied to information processing in the olfactory bulb circuit. They carefully document the transitions from a linear to (multiple) nonlinear regimes with technically impressive experiments. This work is an important contribution to understanding dendritic integration in olfactory bulb granule cells.

1. The abstract could be clearer and "punchier".

Some suggestions: lines 20-21: It's not clear to me that placing this idea of inactivation in the abstract is that critical - a general reader may not be expert enough to appreciate it (of course it should be discussed in the Intro/Methods). Perhaps just say "... two-photon uncaging of glutamate at synaptic resolution, along with..." or some such?

lines 25-26: "until a few spines below global Na+ spike threshold" - this is not clear on initial reading. You mean, of course, until a threshold number of spines are activated… Please clarify. lines 28-29: The final line seems a bit abrupt and doesn't quite follow easily from the earlier parts.

*Many thanks for the suggestions. The abstract has been expanded from 150 to 250 words and reworked accordingly.*

2. The authors could consider beginning the introduction with a more general statement about the importance of dendritic integration for information processing throughout the brain (to increase interest for the general audience) and emphasize that olfactory granule cells are a good model system in which to study this since dendritic integration is directly involved in transmitter release.

*We have added a general introduction on dendritic integration and used it to introduce the various active conductances and associated spike types that contribute to integration in a more general context; we now also introduce dendritic transmitter release from this vantage point (l 38-61).*

3. Line 61: "result" should be "results"

*Fixed*

4. Line 93, figure 1: What's the reason for the unconventional shapes of the MNI-evoked EPSPs in some cases? Some seem to peak, and then have unusual rising phases (rather than the expected decays over 10s of milliseconds)?

*The reason for these apparently slow signals is the high spontaneous activity in our bulb slices which seems to be a general property of the bulbar network (Egger & Urban 2006) and is sometimes further increased in the presence of caged Glu compounds (see our previous study Bywalez et al. 2015). Due to the low number of stimulations per single spine because of recording time restrictions, we only dismissed EPSPs where already the baseline/ rising phase before the first peak was contaminated by spontaneous activity. This procedure has now been clarified in the Methods section (l 854-856).*

5. line 159: TPU is not defined explicitly here, before or later... at least I couldn't find it!

*TPU (two-photon uncaging) as abbreviation has been removed from the manuscript.*

6. Line 172, figure 2c: I am not fond of the color bars highlighting the categories - why not just color the points themselves? Also in figures further on. [Of course, this is perhaps an esthetic choice]

*Thanks for the input! Coloring the points also increased coherence with other figure parts.*

7. Line 198-199: this result is interesting and perhaps the authors can directly state that there could be Ca regenerative events withOUT Na events?

*Indeed, we find here that these Ca2+ spikes are purely dendritic, in contrast to the previously observed global Ca2+ spikes. We have added an explanatory remark to the Results l. 263 ff: ‚The regional dendritic Ca^2+^-spike observed here differs from earlier observations of granule cell global Ca^2+^-spikes (also termed low-threshold spikes) that were generated by glomerular or external electrical field stimulation (17, 19), and that spread evenly throughout the dendrite and also boost and broaden somatic EPSPs (see Discussion).‘*

*We also have extended the Discussion of this observation, including possible explanations for the different observations (e.g. axonal inputs from mitral cells to granule cell basal dendrites during glomerular stimulation), in l. 680-696.*

8. Lines 673-675. The authors write in the methods that "in order to provide optimal optical access to the GC apical dendritic tree, patched GCs were located close to the mitral cell layer (MCL)." It would be helpful to be more specific here. Ideally, if the authors measured the distance from the mitral cell layer for each cell, this should be included. If this was not done, at least an estimate (i.e. <100 μm below the mitral cell layer) would be helpful.

*These data - mean depth below MCL 14 ± 12 µm, n = 63 cells - have been included in the Methods (l 792 f).*

9. Line 492-onwards: Although the authors test other morphological parameters such as spine neck length, we did not see any mention of spine head size. Even if this was not tested as a parameter affecting integration (perhaps it could be tested with data that had already been collected?), it would be helpful to know: how did the authors choose which spines to stimulate? Presumably there was a bias towards larger mushroom spines since filopodial spines would be less easily visualized (although some of the spines in Figure 1a look quite small/filopodial so perhaps this was not the case), but was there a cutoff for the size of the spines chosen? For testing clustered inputs, did the authors choose any spines within the desired distance to stimulate or only large mushroom spines? I realize the authors have already done a lot of regressions, but we are merely curious!

*We very much appreciate this idea, since spine head shapes and sizes are of general interest in the field. However, since our final stacks were not taken at sufficient z-resolution for deconvolution, we had to resort to a rather simple estimate here, using the FWHM of the fluorescence (as in Egger Stroh 2009), now included in the Methods (l 930-932). While there was no correlation, we have included these data now in Table 1 and Figure 8.*

*In general, there is certainly a bias in our selection of spines towards larger spines since filopodial spines often became visible just at the end of experiments (using high laser power for raster scanning). We also have not targeted filopodial spines „on purpose“ in our previous study (Bywalez et al 2015). Due to the lack of z-resolution in our image stacks we did not wish to go deeper into spine shape analysis at this point.*

*With regard to clustering, all our spine sets may be regarded as clustered compared to studies in pyramidal cells due to the holographic FOV of 70 x 70 x 70 µm^3^. On the other hand, since the branching part of a granule cell’s apical dendritic tree is often not larger than 200 µm, criteria for clusters on granule cell dendrites might be different.*

*We did not choose tight or loose clusters on purpose, the choice was rather dictated by the arrangement of available spines, as now mentioned also in Methods l. 841-843; granule cell overall spine density is usually low. Occasionally selected spines were observed to disappear or move, in line with the high structural plasticity reported elsewhere (Sailor et al. 2016, Zhang et al 2016). These observations however were too rare to warrant further mention.*

10. Line 582 onwards: The paragraph in the discussion beginning with "All GCs in our sample featured Ca2+-spikes…" (line 582) is confusing to this reader. Please clarify the relationship of this work with your previous work (ref. 5) with regards to local/global calcium spikes. Does glomerular stimulation give different results than uncaging in terms of localization of calcium signals?

*Yes, it does, the paragraph in question has been reworked to clarify the observation and possible explanations for the difference are discussed in more detail now, such as axonal inputs from mitral cells to granule cell basal dendrites during glomerular stimulation (in l. 680-696). This should also settle point 7 raised above by the same reviewer.*

11. Line 593: "subthreshold" is misspelled

*Corrected.*

12. Rats were postnatal day postnatal day 11-21 - were any differences or trends across age observed? Previous work has shown changes in GC properties across this time period (e.g. Dietz and Murthy 2011).

*We have performed these regressions and found no influence across age; the result is now included in Table 1 and in Fig. 8. The range of analysed cells is actually a little more narrow (P11-P18), but still..*

13. Some of the figures are a bit cluttered, confusing and lack legends on the figure. For example, in Figure 2, it would be clearer to put a legend showing that red = AP and green = Ca2+ and then list the spine numbers activated for each trace on the left or right side of the trace rather than having the numbers below the peaks of the traces where they can be lost in the jumble. Ideally magenta should be used instead of red out of consideration for color blindness. In 2a, what does "SLM Vm" mean (should it just be Vm)? Also, what do error bars in this figure mean? Finally, why are different symbols (diamonds, triangles facing to the left or the right or upwards) used in different subplots)? Perhaps simple dots would suffice for all.

*In the revised figures, more space was added between panels and figure panels were in part rearranged (all parts with Nav pharmacology are now assembled in Fig. 5 and the NMDAR pharmacology is now Fig 6).*

*Legends with color codes were added and spine numbers were moved to the legends.*

*Red was replaced with magenta throughout, with the exception of Fig 9 (but there the intensity also encodes the amount of ∆Ca2+).*

*2a: SLM Vm was referring to multi-site uncaging, and is now omitted/replaced in both Fig 1 and 2.*

*The meaning of error bars in Fig 2 is now denoted.*

*The different symbols were used to facilitate the comparison of the same parameters across figures (e.g. rate of rise is always a classical filled triangle) – the use of these symbols has been further unified, and dots are now used for threshold values.*

14. Please explain Figure 4b, right panel further. Why is the dendrite DF/F below 1 - what is it normalized to? Perhaps it would be clearer to normalize to the average dendrite DF/F for the first for spines as in the left panel of the same figure, unless there is a specific reason to present it differently?

*The reason is that ∆F/F in dendrites for the first four spines is usually barely detectable, thus this normalization would cause a big variance (we have used this method at first). The reason is now explicitly stated in Methods l 911-913; the normalization is also mentioned in the caption of Fig. 4.*

15. A summary panel at the end of Figure 7 showing summation effects for Ca spikes, D-spikes, and global Na spikes could be helpful.

*A graphical summary is now included (new Figure 9).*

*Added references (mostly within the new general introduction and in response to requests by the reviewers):*

*Stuart GJ, Spruston N. Dendritic integration: 60 years of progress. Nat Neurosci. 2015;18(12):1713-21.*

*Larkum ME, Waters J, Sakmann B, Helmchen F. Dendritic spikes in apical dendrites of neocortical layer 2/3 pyramidal neurons. J Neurosci. 2007;27(34):8999-9008.*

*Remy S, Csicsvari J, Beck H. Activity-dependent control of neuronal output by local and global dendritic spike attenuation. Neuron. 2009;61(6):906-16.*

*Makara JK, Magee JC. Variable Dendritic Integration in Hippocampal CA3 Pyramidal Neurons. Neuron. 2013;80(6):1438-50.*

*Kim S, Guzman SJ, Hu H, Jonas P. Active dendrites support efficient initiation of dendritic spikes in hippocampal CA3 pyramidal neurons. Nat Neurosci. 2012;15(4):600-6.*

*Abrahamsson T, Cathala L, Matsui K, Shigemoto R, DiGregorio DA. Thin Dendrites of Cerebellar Interneurons Confer Sublinear Synaptic Integration and a Gradient of Short-Term Plasticity. Neuron. 2012;73(6):1159-72.*

*Payeur A, Beique JC, Naud R. Classes of dendritic information processing. Curr Opin Neurobiol. 2019;58:78-85.*

*Schoppa NE, Westbrook GL. Glomerulus-specific synchronization of mitral cells in the olfactory bulb. Neuron. 2001;31(4):639-51.*

*Halabisky B, Strowbridge BW. γ-Frequency Excitatory Input to Granule Cells Facilitates Dendrodendritic Inhibition in the Rat Olfactory Bulb. Journal of Neurophysiology. 2003;90(2):644-54. doi: 10.1152/jn.00212.2003. PubMed PMID: 12711716.*

*Kishi K, Mori K, Ojima H. Distribution of local axon collaterals of mitral, displaced mitral, and tufted cells in the rabbit olfactory bulb. J Comp Neurol. 1984;225(4):511-26. doi: 10.1002/cne.902250404. PubMed PMID: 6203939.*

*Pinato G, Midtgaard J. Regulation of granule cell excitability by a low-threshold calcium spike in turtle olfactory bulb. J Neurophysiol. 2003;90(5):3341-51. doi: 10.1152/jn.00560.2003. PubMed PMID: 12867531.*

*Urban NN, Sakmann B. Reciprocal intraglomerular excitation and intra- and interglomerular lateral inhibition between mouse olfactory bulb mitral cells. J Physiol. 2002;542(Pt 2):355-67.*

*Mehrke G, Zong XG, Flockerzi V, Hofmann F. The Ca(++)-channel blocker Ro 40-5967 blocks differently T-type and L-type Ca++ channels. J Pharmacol Exp Ther. 1994;271(3):1483-8.*

*Kelsch W, Lin CW, Lois C. Sequential development of synapses in dendritic domains during adult neurogenesis. Proc Natl Acad Sci U S A. 2008;105(43):16803-8.*

*Dietz SB, Markopoulos F, Murthy VN. Postnatal development of dendrodendritic inhibition in the Mammalian olfactory bulb. Front Cell Neurosci. 2011;5:10.*

*McTavish TS, Migliore M, Shepherd GM, Hines ML. Mitral cell spike synchrony modulated by dendrodendritic synapse location. Front Comput Neurosci. 2012;6:3.*

*Cazakoff BN, Lau BY, Crump KL, Demmer HS, Shea SD. Broadly tuned and respiration-independent inhibition in the olfactory bulb of awake mice. Nat Neurosci. 2014;17(4):569-76*

*Youngstrom IA, Strowbridge BW. Respiratory modulation of spontaneous subthreshold synaptic activity in olfactory bulb granule cells recorded in awake, head-fixed mice. J Neurosci. 2015;35(23):8758-67.*

*Losacco J, Ramirez-Gordillo D, Gilmer J, Restrepo D. Learning improves decoding of odor identity with phase-referenced oscillations in the olfactory bulb. Elife. 2020;9.*

*Bevington PR, Robinson DK. Data Reduction and Error Analysis for the Physical Sciences. 3^rd^ edition. 2003, McGraw-Hill, New York.*

*Egger V, Stroh O. Calcium buffering in rodent olfactory bulb granule cells and mitral cells. J Physiol. 2009;587(Pt 18):4467-79.*

*After submission, there has been one major study on dendritic integration, concerning retinal ganglion cell subtypes (Ran… Euler, Nat Comm, April 2020). However, there is little overlap both in the methods and findings with our study. Since we are planning to put our results in context with findings in the retina in a review with Jeff Diamond, we did not wish to embark on such a comparison in the current manuscript.*
