# Supplementary material for: Dendritic integration in olfactory bulb granule cells upon simultaneous multispine activation: Low thresholds for nonlocal spiking activity
Source: PLoS Biol. 2020 Sep 23;18(9):e3000873. doi: 10.1371/journal.pbio.3000873 (PMC7535128; doi:10.1371/journal.pbio.3000873)
Supplement: Attachment — Submitted filename: ResponseToReviewers.docx [file pbio.3000873.s005.docx]

*We wish to thank the reviewers for their appreciation of our efforts to improve the manuscript. We also wish to state again our gratefulness for the detailed suggestions made by the reviewers.*
